# The expansion of Acheulean hominins into the Nefud Desert of Arabia

**DOI:** 10.1038/s41598-021-89489-6

**Published:** 2021-05-12

**Authors:** Eleanor M. L. Scerri, Marine Frouin, Paul S. Breeze, Simon J. Armitage, Ian Candy, Huw S. Groucutt, Nick Drake, Ash Parton, Tom S. White, Abdullah M. Alsharekh, Michael D. Petraglia

**Affiliations:** 1 Pan-African Evolution Research Group, Max Planck Institute for the Science of Human History, Kahlaische Strasse 10, 07745 Jena, Germany; 2 Department of Classics and Archaeology, University of Malta, Msida, Malta; 3 Institute of Prehistoric Archaeology, University of Cologne, 50931 Cologne, Germany; 4 Department of Geosciences, Stony Brook University, Stony Brook, NY 11794-2100 USA; 5 Turkana Basin Institute, Stony Brook University, Stony Brook, NY 11794-4364 USA; 6 Research Laboratory for Archaeology & the History of Art, School of Archaeology, University of Oxford, Oxford, OX1 3TG UK; 7 Department of Geography, King’s College London, 40 Bush House (North East Wing), Aldwych, London, WC2B 4BG UK; 8 Centre for Quaternary Research, Department of Geography, Royal Holloway University of London, Egham, TW20 0EX Surrey UK; 9 SFF Centre for Early Sapiens Behaviour (SapienCE), University of Bergen, Post Box 7805, 5020 Bergen, Norway; 10 Extreme Events Research Group, Max Planck Institutes for Chemical Ecology, The Science of Human History, and Biogeochemistry, Hans-Knöll-Strasse 8, 07745 Jena, Germany; 11 Department of Archaeology, Max Planck Institute for the Science of Human History, Kahlaische Strasse. 10, 07745 Jena, Germany; 12 Human Origins and PalaeoEnvironments Research Group, Oxford Brookes University, Oxford, OX3 0BP UK; 13 Mansfield College, University of Oxford, Mansfield Rd, Oxford, OX1 3TF UK; 14 Department of Life Sciences, The Natural History Museum, Cromwell Road, London, SW7 5BD UK; 15 Department of Archaeology, College of Tourism and Archaeology, King Saud University, Riyadh, Saudi Arabia; 16 School of Social Science, University of Queensland, Brisbane, QLD 4072 Australia; 17 Human Origins Program, National Musuem of Natural History, Smithsonian Institution, Washington, DC 20560 USA; 18 Australian Research Centre for Human Evolution (ARCHE), Griffith University, Brisbane, QLD Australia

**Keywords:** Archaeology, Palaeoecology

## Abstract

The Arabian Peninsula is a critical geographic landmass situated between Africa and the rest of Eurasia. Climatic shifts across the Pleistocene periodically produced wetter conditions in Arabia, dramatically altering the spatial distribution of hominins both within and between continents. This is particularly true of Acheulean hominins, who appear to have been more tethered to water sources than Middle Palaeolithic hominins. However, until recently, chrono-cultural knowledge of the Acheulean of Arabia has been limited to one dated site, which indicated a hominin presence in Marine Isotope Stages (MIS) 7–6. Here, we report the first dated Acheulean site from the Nefud Desert of northern Saudi Arabia, together with palaeoecological evidence for an associated deep, probably fresh-water, lake. The site of An Nasim features varied and often finely flaked *façonnage* handaxes*.* Luminescence ages together with geomorphological and palaeoecological evidence indicates that the associated artefacts date to MIS 9. At present, An Nasim represents the oldest yet documented Acheulean sites in Arabia, and adds to a growing picture of regionally diverse stone tool assemblages used by Middle Pleistocene hominins, and likely indicative of repeated population re-entry into the peninsula in wet ‘Green Arabia’ phases.

## Introduction

The Acheulean was a long-lasting and geographically widespread hominin technology used in many regions across the Old World. Distinctive Acheulean stone tool assemblages are characterized by the production of large cutting tools dating from ~ 1.7 million years ago^1,2^ until as late as 130 thousand years ago (ka) in some places^3,4^. The Acheulean is frequently described as technologically homogenous, lacking in variation in comparison with subsequent techno-cultural phases—a fact that runs counterintuitively to its vast temporal and spatial distribution, and the probability that it was the product of more than one hominin species^4–7^. Despite this general view and in the absence of hominin fossils, it has been possible to link population turnovers with material culture diversity in some regions, particularly in glaciated or dryland regions where hominin occupations are strongly modulated by palaeoenvironmental factors^8,9^.

The repeated expansion and contraction of the mid-latitude deserts present one such major biogeographic constraint, in this case to dispersals between and within Africa and Southwest Asia. Periodic environmental amelioration, including increased rainfall, transformed current regions of hyper-arid desert into grasslands with extensive networks of rivers, wetlands and lake^10–13^. The Arabian Peninsula in particular is situated at a critical nexus within the Saharo-Arabian arid belt, and research has demonstrated that this region experienced dramatic environmental oscillations that periodically transformed ecological and hydrological barriers across continents^11,14–17^. The diachronic patterns of the Acheulean here therefore have the potential to provide much-needed insights into the links between regional population turnover between Africa and different parts of Southwest Asia, and to cast light on the changing character of hominin landscape behaviours over time.

Despite its clear geographic importance, detailed knowledge of the Acheulean in Arabia is currently limited to a single well-documented site. Saffaqah, in central Saudi Arabia is situated beside an andesite dyke that was heavily exploited for its desirable raw material^18–20^. Here, minimally trimmed and often asymmetric handaxes were made on large flakes struck off giant andesite cores. Despite the seemingly unrefined appearance of these handaxes, they date to the later part of Marine Isotope Stage (MIS) 7 (~ 243–192 ka), and seemingly persisted into MIS 6^18^. Elsewhere in Arabia, however, highly symmetrical and finely trimmed *façonnage* handaxes have been reported, particularly from palaeolake deposits in the Nefud Desert of northwest Saudi Arabia^21^. These are diverse lithic assemblages, ranging from Micoquian-type handaxes to a variety of triangular, sub-triangular and ovate forms ranging in size from ~ 7 cm in length to ~ 20 cm or more^21^. On the basis of their fine-flaking and symmetrical forms, the smallest handaxes were hypothesised to be late, perhaps even representing a transitional phase between the Lower and Middle Palaeolithic^21^. While their riverine and lacustrine associations point to significantly wetter conditions, until recently, no handaxe assemblages in the Nefud Desert were closely associated with dated sediment deposits that permitted chronometric assignment. Furthermore, a wide diversity of palaeolake deposits exist, and vary in terms of their age and inferred size and water depth, though few have been studied in detail^13,21–23^. As a result, little is known about the local climatic and environmental conditions that prevailed during different humid phases in a ‘Green Arabia’, and the extent to which they influenced the presence and behaviour of hominin populations.

**Figure 1:**
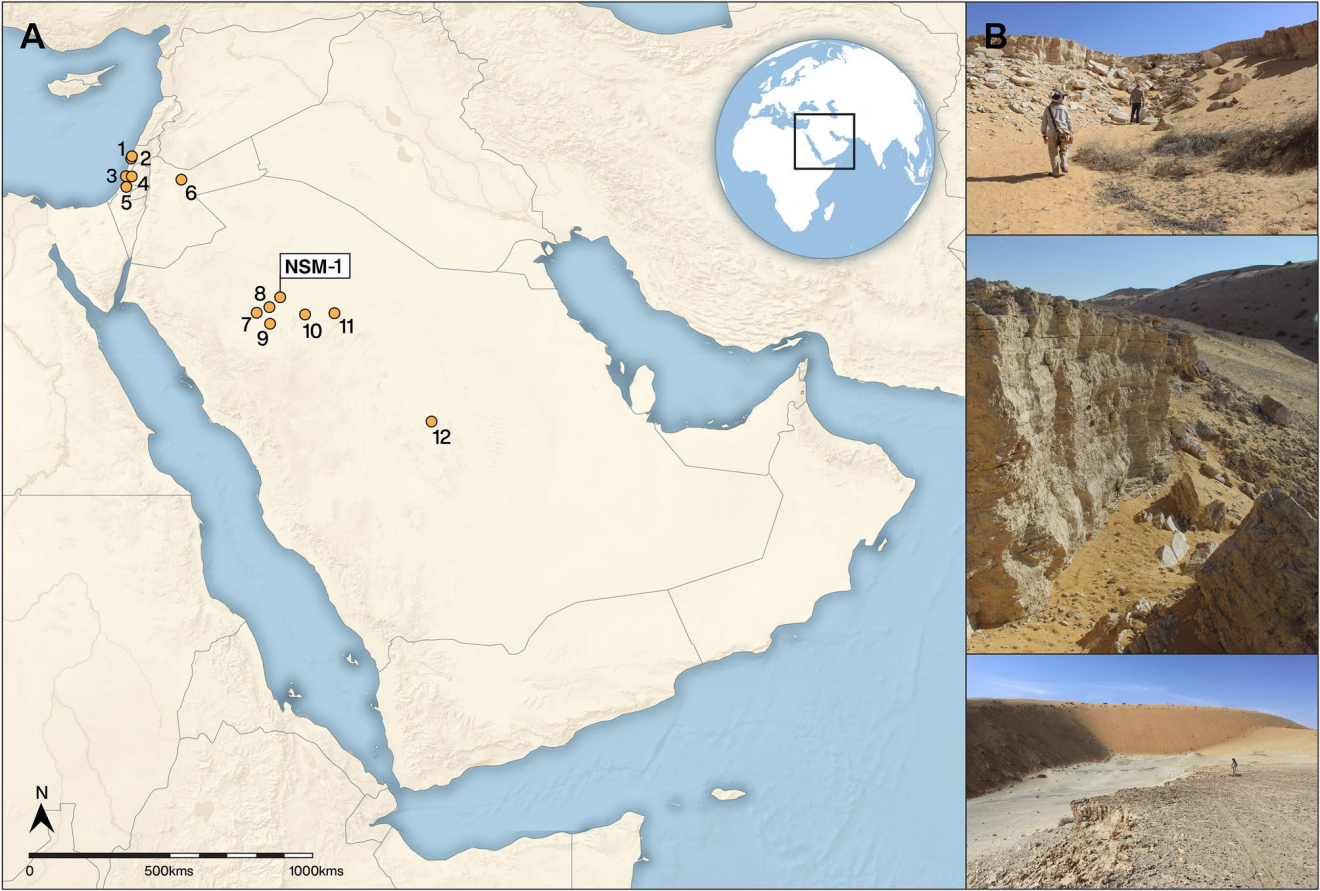
Map of Arabia with the site of An Nasim (NSM-1) and key Levantine and Arabian sites, including those discussed in the text. Tabun (1), Misliya (2), Holon (3), Qesem (4), Revadim (5), Azraq (6), Khabb Musayyib-2 (7)^21^, Khall Amayshan-1 (8)^21^, Ti’s al Ghadah (9)^25,38,39^, Al Marrat-6 (10)^15,40^, Al Qana-1 (11)^15,41,42^, Saffaqah (12)^18,19,43^. The map was created using QGIS 3.12 https://qgis.org/en/site/ and the Natural Earth Database from https://www.naturalearthdata.com/downloads/ and Adobe Illustrator CC.

Here, we report the first dated Acheulean site from the Nefud Desert of northern Arabia. The An Nasim site was discovered using remote sensing and palaeohydrological modelling^11,15^ by the Palaeodeserts Project in 2015. The site is associated with a particularly thick (> 4 m) palaeolake deposit in an area where other lakes have been dated to MIS 9 (~ 337–301 ka)^23^. The An Nasim locality is an interdune basin approximately 20 km from the western edge of the western Nefud dune field, in the Ha'il province of Saudi Arabia (Figs. 1, S1). Bounded by north–south oriented transverse barchanoid mega-dunes, the basin contains a series of marl deposits indicative of former lake phases (Fig. S1). This setting is typical of the Middle and Late Pleistocene record of much of the Nefud^13^, where interdune depressions that formed during arid phases provide accomodation space within which lakes and associated sediments form during humid periods. An Nasim is one of a large number of such basins containing palaeolake deposits within the Western Nefud^11,13,21,23^, but it is atypical in terms of the thickness of the deposits present within the basin (Fig. S1)—for contrast see^24^, and^13^. However, remote sensing analyses^13^ indicate this to be characteristic of the An Nasim region, where several other local basins also exhibit thick sequences. Below, we provide a detailed description of the palaeotopography, dating and geomorphology of the Middle Pleistocene parent basin and lake, along with the chronology and character of the lake's Acheulean artefacts, considering their comparative place in the broader Arabian Acheulean.

## Results

**Figure 2:**
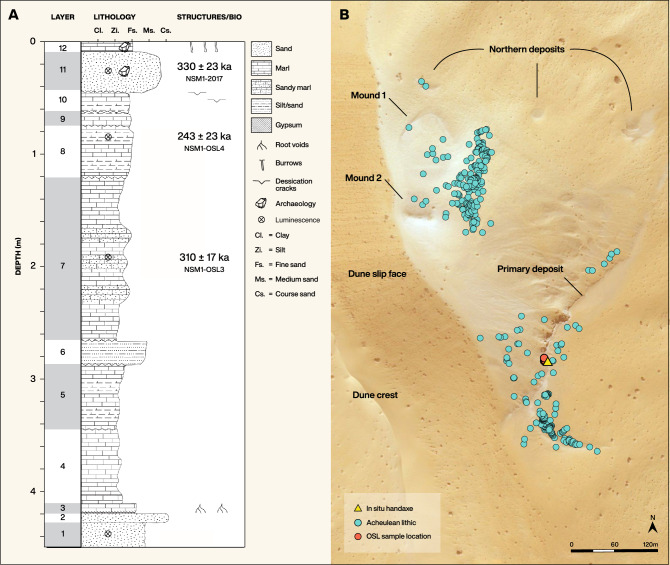
Stratigraphic sequence of An Nasim and artefact distributions. (**a**) stratigraphy with the locations of the sediment samples dated by luminescence; (**b**) Lower Palaeolithic artefacts at An Nasim, mapped through systematic survey of the current interdune and recorded using a differential GPS system. The stratigraphic sequence was drawn from the location of the handaxe in Layer 12. Produced using ArcMap version 10.2. Basemap from Bing Maps Aerial, (**c**) 2010 Microsoft Corporation and its data suppliers.

**Figure 3:**
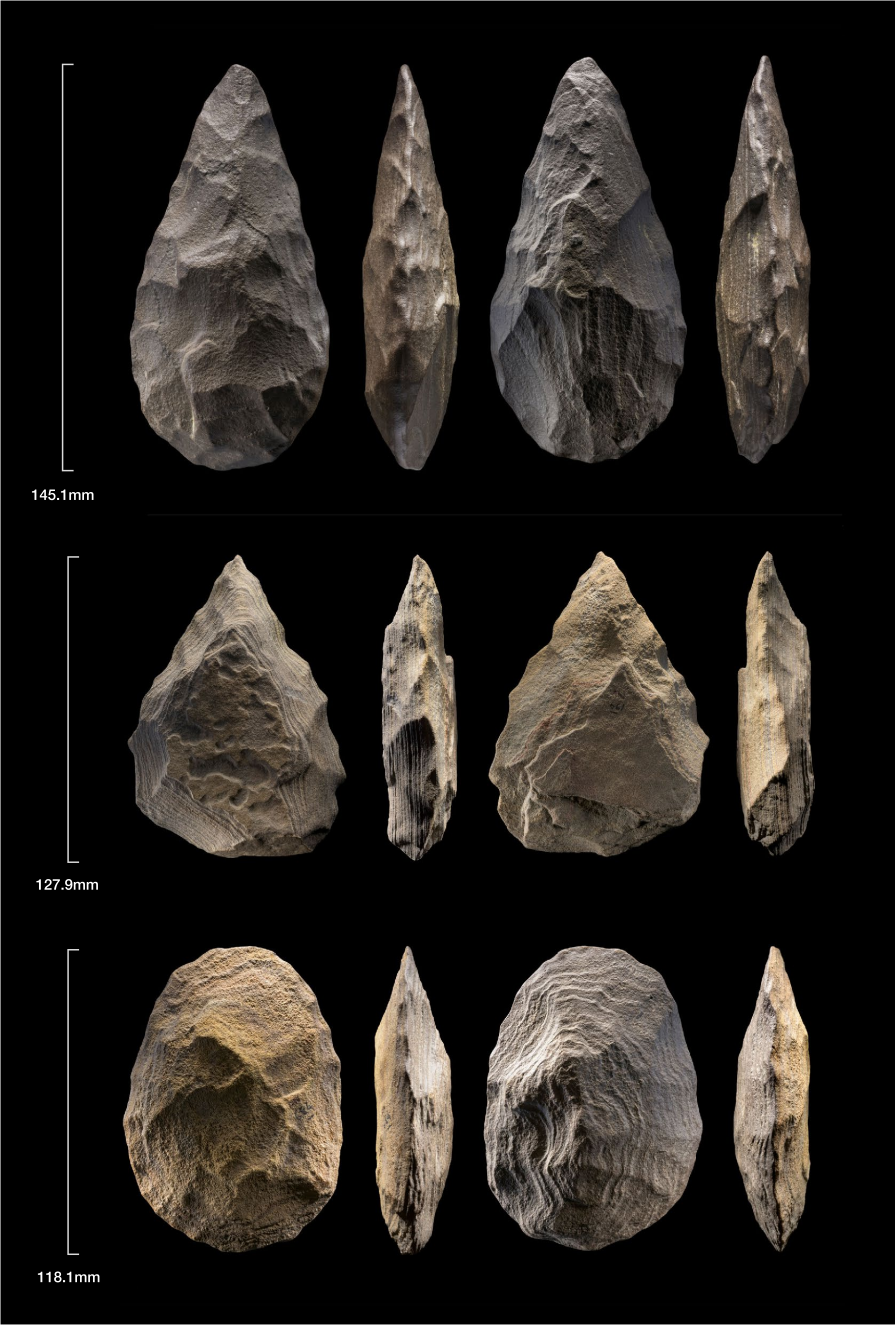
Different handaxe forms from An Nasim. Credit: Ian Cartwright.

An Nasim consists of deep and narrow interdunal basin in which a sequence of aeolian sands overlain by bedded lacustrine marl is preserved (Figs. 2, S1). In the central part of the An Nasim basin, outcrops of these deposits are exposed extending approximately 800 m north–south and 350 m east–west. The marl outcrops are, however, fragmented and discontinuous, occurring at several distinct altitudes (Fig. S1). The thickest visible exposures of marl are found along the basin's eastern edge (Figs. 2, 3, S1). At the base of these exposures, the deposits express the morphology of the former interdune depression in which they accumulated, in the form of a concave surface dipping steeply away from the edge of the observable outcrops towards the centre of the basin within which they formed. The stratigraphy of the deposits also dips towards the centre of this palaeobasin, indicative of sediments being deposited in a quiescent water body and draping across the existing topography. The western edge of the deposit is at ~ 930 m above sea level (MASL) and has been deeply eroded, forming a small cliff (maximum of 4 m high) providing a thick exposure of lake sediments. Large ‘boulders’ of sediment at the base of this cliff have been dislodged and transported down-slope towards the centre of the current interdune depression. The marls are thickest at the western edge, which likely lay towards the centre of their contemporary interdune palaeobasin, and thin in an easterly direction towards its edges (0.5 m at their thinnest). The thickness of the marl deposits in the central area is exceptional in comparison to previously excavated comparable late Middle and Late Pleistocene deposits found elsewhere in the western Nefud^22,24,25^. An additional area of palaeolake deposit exists immediately to the south of the primary exposure at the same altitude, likely a continuation of the same deposit in an area that has experienced differential erosion.

The undulating lower contact and complex bedding geometry of the lake sediments reflect the accumulation of these sediments over a pre-existing aeolian dune topography. In this context, the marl sediment precipitates from the water column, falls out of suspension and, consequently, accumulates in thick beds that drape over the sand dune forms that are preserved on the lake bed. These beds consequently dip into the centre of the basin and undulate throughout the exposure. The dip of the marl beds means that units that occur several meters below the surface at the western section edge are found at the land surface on the eastern basin margin. Of particular relevance to this study is the fact that the marl-rich sand bed that is found near the surface of the marl unit at the outcrop edge, containing lithics in stratigraphic position, can be traced laterally and is found to occur 3 m below the surface towards the centre of the basin (Fig. 3).

The massive marl beds at the base of the section (Fig. 2a) indicate deep water conditions, while towards the top of the sequence the interdigitation of beds of marl and sand, with associated desiccation cracks, are typical of a shallower water body that experienced episodic drying (Figs. 2a, S1). The upper layers 11 and 12 are laterally extensive and contain lithics in stratigraphic position within horizontally bedded sands (Layer 11) overlain by a thin bed of marl (Layer 12—Fig. S1). This sequence suggests falling water level and sheet wash deposition of sands from the surrounding landscape, followed by a small subsequent rise in water level. The sedimentology of the upper part of the primary marl sequence, and in particular that of unit 11, within which a stratified lithic was found, is therefore consistent with the occupation of the site during a drier phase featuring low lake levels.

In arid environments, where reworking is widespread, it is often difficult to demonstrate that lithic artefacts are contemporaneous with the age of the deposit. However, at An Nasim, three observations are important. Firstly, that diagnostic artefacts have been recovered from within the marls and can therefore be directly related to specific strata. Secondly, the size of the lithics (pebble/cobble) is significantly coarser than the grain size of any of the sediments within the host deposits, which are dominated by sands and silts. This observation demonstrates that the processes responsible for depositing these sediments were incapable of transporting and reworking the artefacts. Finally, the surface of the main marl bed is the highest point at the site, meaning that there are no older, higher deposits from which the lithics can be eroded and redeposited in the marl sequence. When these observations are considered the most likely source of the lithics that are found across the surface of the marl bed is the uppermost layers of this unit where stratified archaeology has been directly recovered.

At lower altitudes within the current interdune area, additional marl deposits are visible, all of which are much less distinct and appear more degraded than the primary deposit discussed above. Three small exposures of marl exist on the northern flank of the basin between approximately 930 and 923 MASL, potentially peripheral exposures of the massive marls, whilst at the basin centre two distinct large mounds of eroded marl material are present. Mound 1, the northernmost of these, has a curved upper surface, again suggestive of a lake bed deposited in an interdune basin, this time at around 921 MASL (Fig. 2b). Mound 2 (Fig. 2b), to the south, has an indistinct heavily eroded upper surface at ~ 916 MASL, while its relationship to Mound 1 (Fig. 2b) is unclear. Both are eroded, preserved as inverted relief features above the current interdune floor (which lies at 910 MASL) possessing flanks covered with the deflated remnants of the palaeolake deposits. The stratigraphic relationship of these lower deposits to the primary deposit remains unclear due to deflation having created an unconformity between them. However, the morphology of Mound 1, and the lower altitude of these sediments relative to the primary deposit, strongly indicates that they belong to a lacustrine phase distinct from that of the primary deposit. It is likely that they formed in the floor of a later interdune depression, prior to the more recent deflation that created the present interdune area that they lie within. An Nasim thus preserves several discrete phases of lake basin development separated by episodes of aeolian deflation related to cyclic climate change within the western Nefud.

The sedimentological observations at An Nasim are in keeping with the picture observed across the wider western Nefud Desert, where the repeated raising of regional groundwater levels during discrete humid intervals produced lakes and wetlands in the interdune depressions^13,24^. Previous analyses have indicated the these palaeolakes were widespread across the western Nefud, and that despite an absence of evidence for large-scale fluvial activity within the region, the high density of such interdune lakes facilitated hominin dispersals through it^11,13^.

At An Nasim, two discrete concentrations of Lower Palaeolithic artefacts were discovered distributed across the surfaces of the primary deposit, and the lower mounds (Fig. 3). Systematic collection recovered 354 artefacts, primarily handaxes, together with various flakes that included clearly identifiable bifacial thinning flakes (Table 1). The artefacts were found in two main clusters at the site (Fig. 2b) and appear to be eroding out of the marl deposits. All visible artefacts were systematically collected and their locations recorded using a differential GPS (DGPS). However, it should be noted that ever-shifting sands likely hid other artefacts from view, and were therefore not collected. We acknowledge that the assemblage may therefore be biased towards handaxes, which are larger and thicker and therefore less easily buried than flakes. The results of this survey, mapped in Fig. 3, illustrate the close association between the artefacts and the lake.

**Table 1:** Breakdown of artefact classes from An Nasim. Flake numbers are likely an underestimate from the site, as shifting sands hid smaller artefacts from view.

Artefact type	Number
Handaxe	286
Flakes, including bifacial thinning flakes	68
Discoidal core	4

The lithic tools are similar to previously reported Acheulean sites in the Nefud Desert^21^ and consist of relatively thick and finely flaked bifaces (typically triangular and pointed). The artefacts represent the entire bifacial manufacturing sequence, all of which were constructed by thinning out large tabular blocks of ferruginious quartzitic sandstone^26^. The presence of minimally flaked pieces of these tabular blocks indicate that the raw material was brought to the site, some of it apparently discarded after having been ‘tested’ by the removal of one or two flakes along an edge. Other flaked pieces were very roughly shaped before being abandoned. Many of the handaxes retained the last vestiges of the flat, tabular cortical surface at their centre, often on both faces. The base of the handaxes also frequently retained the thick, flat cortical edge of the tabular block, perhaps to aid grasping. None of the bifaces were made from flakes and there was no evidence of large flake manufacture, perhaps due to the small, tabular nature of the local raw material. Indeed, broader surveys in the Nefud Desert indicate that this local tabular quartzite was frequently used at other undated Acheulean surface assemblages, all of which lacked evidence for large flake manufacture^21^. This suggests the local raw material impeded this approach to handaxe manufacture.

**Figure 4:**
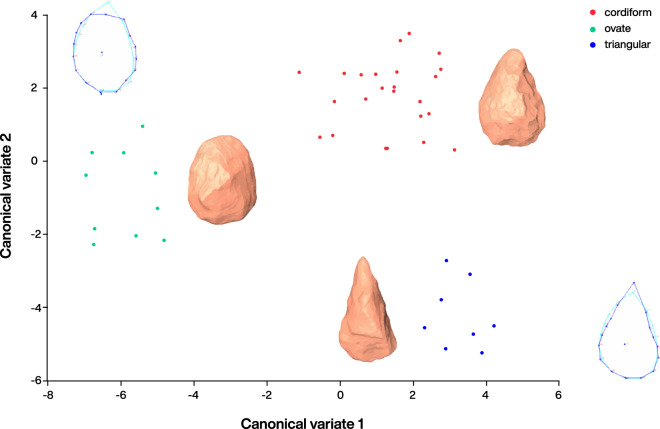
Canonical Variates Analysis of Biface form (n = 50) at An Nasim, showing discrete shape groupings corresponding to triangular, ovate and cordiform forms. See Tables S2–S4 for eigenvalues and distances.

The surface artefacts exhibited a similar high degree of weathering, while the artefacts from buried or recently exposed contexts were fresh. The handaxes were diverse in form, ranging from ovate to cordiform and triangular forms, as at other Acheulean sites in the Nefud^21^, and variable in size (Fig. 3). All handaxes with observable flake scars showed fine flaking, regardless of form. 2D Geometric Morphometric (GMM) analysis of a random sub-sample of fifty handaxes showed that this form variation was not continuous (Figs. 4, S1, Tables S2–S4). However, no spatial relationship between discrete forms and findspots was observed in the sample.

Survey revealed one face of a stratified handaxe visible in the section in the top 10 cm of the primary marl deposit (Layer 12—Fig. S1). Small-scale excavation in the form of a shallow 1 × 1 m test trench around this location allowed the subsequent recovery of this firmly embedded handaxe. This handaxe was included in the 2D GMM analysis shown in Fig. 3, where it clustered with the cordiform group found on the surface. The tight, shape-based clustering of the cordiform handaxes, along with the similarity of manufacture and raw material indicates that these forms at least, may be regarded as contemporary with each other in the marl. The similarity of manufacture among all the handaxe forms represented at An Nasim may also indicate broad contemporaneity. Digging for a sediment sample for dating purposes also permitted the recovery of a bifacial thinning flake cemented within the sandy Layer 11.

A sample for luminescence dating was collected from Layer 11 (NSM1-2017), where archaeology was also recovered (Fig. 2a, See SI), and additional samples were collected beneath the lithic horizon in Layer 8 (NSM1-OSL4) and Layer 7 (NSM1-OSL3). Dose rates for these samples were determined by thick source alpha and beta counting, while gamma dose rates were measured using a field gamma spectrometer (See Table 2, SI, Table S5).

**Table 2:** IR-RF age results.

Sample ref.	Layer	#^a^	De^b^ (Gy)	OD (%)	Dr (Gy ka^−1^)	Age^c^ (ka)
NSM1-2017	11	6/6	520.2 ± 25.7	11 ± 3	1.58 ± 0.08	330 ± 23
NSM1-OSL4	8	5/10	441.6 ± 37.2	17 ± 6	1.82 ± 0.08	243 ± 23
NSM1-OSL3	7	4/10	516.9 ± 14.7	4 ± 2	1.67 ± 0.08	310 ± 17

^a^Number of aliquot giving an IR-RF signal/measured.

^b^Unweighted mean ± se.

^c^Calculated using DRAC v.1.2^28^, given at 1 sigma.

The K-feldspar grains were isolated and then analysed using the infrared-radiofluorescence protocol at controlled temperature (RF_70_) (See SI 3)^27^, using the same parameters as described previously^19^. IR-RF dose and age estimate are reported in Table 1. The overdispersion values (OD) are less than 20%, which is consistent with our prediction for such sediment. The three samples yield ages of 310 ± 17 (NSM1-OSL3), 243 ± 23 ka (NSM1-OSL4) and 330 ± 23 ka (NSM1-2017). These ages are coherent at 2 sigma, however sample NSM1-OSL4 is much younger than the other two samples, which yield very similar ages. The two older ages also have lower overdispersion values than the younger one, possibly suggesting that they are more reliable.

**Figure 5:**
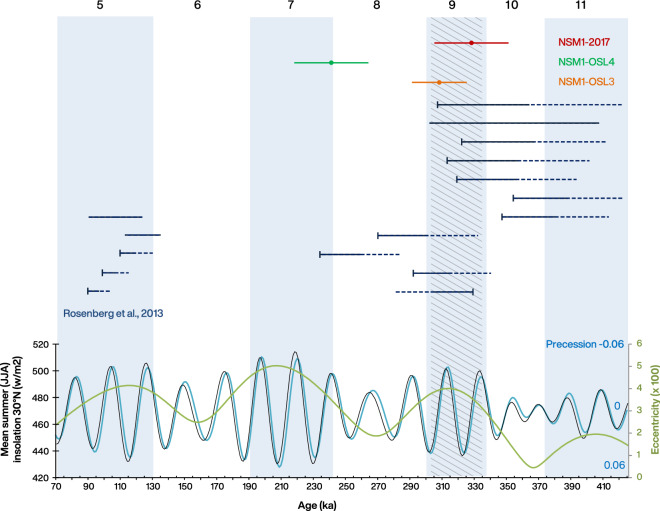
Luminescence ages from the An Nasim site, displayed above the orbital parameters (derived from^44^) which produced humid episodes in the Arabian Peninsula (eccentricity [green] modulation of precession [turquoise], with a corresponding influence upon summer [JJA] insolation at the latitude of the Nefud [black], driving monsoon incursion). Marine Isotope Stages of the last 700 ka are displayed for reference. Navy blue bar data are from^23^ and are displayed as follows. Solid bars indicate lake formation occurred during this range (a direct date or paired bracketing ages). Dashed lines with endcaps and thick bars to the left indicate maximum (underlying, no unconformities) ages for lake formation—which likely occurred either before (i.e. older than) the endcap, or during the period denoted by a thick bar. Dashed lines with endcaps and thick bars to the right indicate minimum (overlying, no unconformities) ages for lake formation—which likely occurred after (i.e. younger than) the endcap, or during the period denoted by a thick bar. The hashed area shows the high concurrence of data suggesting lake formation in MIS 9. Produced using Microsoft Excel.

To further contextualize these age determinations, we compared the ages with mean summer insolation at the latitude of the Nefud Desert, (Fig. 5), the driver of ‘Green Arabia’ humid phases^14^. The buried handaxe is associated with a thick marl sequence overlying the dated sediments. Sedimentological analysis indicates these marls were produced by significant wet conditions. Both the MIS 9 and MIS 7 insolation peaks are modulated by high eccentricity (Fig. 5) and are equal or greater in intensity to that of MIS 5a, which is known to have been wet enough to enable large perennial deep lake formation^24^. As can be seen, the MIS 9 insolation peaks lie closest to the older age estimates and correspond to a time when other lakes in the An Nasim area are known to have formed^23^ (Fig. 5). Taken together, this evidence is consistent with a MIS 9 date for the formation of the An Nasim deposits, though the possibility a younger MIS 7 age cannot be completely discounted.

## Discussion

The Acheulean assemblage at An Nasim is late Middle Pleistocene in age, dating to between ~ 350–250 ka, likely corresponding with the MIS 9 interglacial, when palaeolake formation was seemingly widespread in the Nefud Desert^23^ (Fig. 5). The sedimentology of the An Nasim marls indicates the presence of a deep lake that would have required substantial rainfall and a rise in the regional groundwater table. Luminescence dates from within and beneath the occupation layers indicate that the lake formed during MIS 9, providing good habitation conditions deep into the Nefud Desert. The Acheulean assemblage is associated with the final stages of the primary lake deposit, which extended across most of the current basin. Acheulean artefacts are found within and on top of the palaeolake sediments, with the latter resulting from the deflation of the archaeology-bearing upper marl deposits.

The similarity between the Acheulean material from An Nasim and other undated Acheulean sites in the Nefud Desert indicates that the palaeolakes of this region provided an important corridor for hominin expansions and a viable habitation network for hominins and by inference, other mammals^11,13,21^. Notably, the technological character of the Acheulean assemblages in the Nefud Desert appears to contrast with the younger Acheulean assemblages at Saffaqah, in central Arabia^18,19^. Unlike the minimally trimmed large flake handaxes from Saffaqah, the *façonnage* handaxes from An Nasim are finely made and variable in size, featuring commonalities in manufacturing techniques, degree of symmetry and refinement. These features, together with the similar degree of weathering and spatially delineated concentrations of the An Nasim finds suggests an occupation of limited duration at An Nasim.

Technological differences observed between An Nasim and Saffaqah may relate to variations in raw material and site activity—Saffaqah is located at a raw material procurement source featuring giant andesite blocks, where primary flaking took place. However, differences in chronology and geographic distances between An Nasim and Saffaqah suggest that the observed material culture variations between these two Acheulean sites may also reflect different handaxe-using populations, or perhaps even species. Partial or complete depopulation of the interior of Arabia likely occurred at the onset of the MIS 8 glacial, given the dominant pattern of regional hyperaridity during such phases. Hominins with Acheulean technology may have repeatedly dispersed southwards from the southern Levant, consistent with observations that palaeohydrological corridors repeatedly facilitated such movements^11^. However, this hypothesis can only be tested when further dated Acheulean sites from the Nefud Desert and southern Levant become available. Late Acheulean sites in the Levant, such as Holon, Revadim and at Azraq (Fig. 1) occur in the approximately 500 to 200 ka time range (see e.g.^4,29–32^). The dating of many of these sites is rather poor, as discussed by Dennell ^33^. In the Levant north of Jerusalem and the Dead Sea, the rather different technology of ‘Acheulo-Yabrudian’ assemblages occur between approximately 400 to 200 ka time range at sites such as Misliya and Qesem (e.g.^34–36^). The Levantine evidence points to high levels of technological variability in the late Middle Pleistocene. Handaxe manufacture occurs in variable frequencies and methods, and handaxes are almost absent from some sequences (e.g.^34^). Within Acheulean assemblages, there are variable levels of core and flake technology (e.g.^37^). The late Middle Pleistocene technological diversity we have uncovered within Arabia—with An Nasim showing a different kind of technology to Saffaqah—further adds to this picture of Southwest Asian technological variability in this time period. A systematic discussion of late Middle Pleistocene technology of regions further afield, such as the northern Levant or eastern Africa, is beyond the scope of this paper. However we emphasise that that the record appears to be highly varied, and in the case of the late Acheulean, often poorly dated. It is therefore difficult to synthesize the evidence simply, using currently available data. Elucidating the meaning of this variability, which likely relates to both demographic and pragmatic factors (e.g. raw material differences), remains an important goal for future research.

Within Arabia, the presence of deep, stable freshwater water bodies in the Nefud Desert during MIS 9, such as at An Nasim, would have facilitated hominin expansions, by providing reliable fresh water sources, and associated mammalian prey and other food sources. The presence of diverse, small to large mammals is evident at interglacial palaeolakes in the Nefud, indicative of expansions of animals into the region during wet phases and illustrating the availability of fauna as dietary resources at watering holes^22,25,38^. The emerging palaeoenvironmental and behavioural evidence highlights the distinct character of the Arabian Acheulean. An Nasim and Saffaqah both demonstrate two distinct forms of Acheulean assemblage in the late Middle Pleistocene of Arabia, both of which differ from the pene-contemporaneous Acheulo-Yabrudian and Late Acheulean in the Levant and the Late Acheulean in Africa^18–20^. Behaviourally flexible Acheulean populations may have fostered their own cultural signatures in the context of Arabia’s location and ecological conditions. This suggests that Arabia should not simply be conceived of as an 'empty space' for hominin populations to move into.

## Conclusions

This paper has presented a new Acheulean lake basin site in northern Arabia. The main stratigraphic sequence of the site relates to a single, continuous climatic cycle of lake formation and disappearance, with the artefact-bearing upper part reflecting the intermittent desiccation of the lake. This lake’s main sequence dates to between ~ 350 and 250 ka, with an age of 330 ± 23 ka coming from layer 11, which contained artefacts. This layer is overlain by a marl layer 12, which also contained artefacts, and which clearly relates to the underlying sediments in terms of a single climatic cycle. We have argued for an MIS 9 age on the basis of lower overdispersion values for dates at the older end of this range, the sedimentology, and the fact that multiple other lake basins in the area have been dated to within MIS 9^23^. The site features finely made, *façonnage* handaxes in a range of forms, all made from large, tabular blocks of ferruginious quartzitic sandstone. These artefacts resemble those from other, undated sites in the same region of the Nefud Desert. These artefacts are unique in Southwest Asia for this timeframe, whether at the younger or the older end of the range, indicating that the Arabian Peninsula was home to a distinctive regional Acheulean that may reflect the particular environmental and demographic conditions of the Peninsula.

## Supplementary Information

Supplementary Information.
