# Supplementary material for: The expansion of Acheulean hominins into the Nefud Desert of Arabia
Source: Sci Rep. 2021 May 12;11:10111. doi: 10.1038/s41598-021-89489-6 (PMC8115331; doi:10.1038/s41598-021-89489-6)
Supplement: Supplementary file 1 — Supplementary Information. [file 41598_2021_89489_MOESM1_ESM.doc]

**The expansion of Acheulean hominins into the Nefud Desert of Arabia**

Eleanor M.L. Scerri^1-2*^, Marine Frouin^3-5^, Paul S. Breeze^6.^, Simon J. Armitage^7-8^, Ian Candy^7^, Huw S. Groucutt^9-11,12^, Nick Drake^6,12^, Ash Parton^13-14^, Tom S. White^15^, Abdullah Alsharekh^16^, Michael D. Petraglia^12,17,18*^

^1^ Pan-African Evolution Research Group, Max Planck Institute for the Science of Human History, Kahlaische Straße 10, 07745, Jena, Germany.

^2^ Department of Classics and Archaeology, University of Malta, Msida, Malta.

^3^ Department of Geosciences, Stony Brook University, Stony Brook, New York 11794-2100, USA.

^4^ Turkana Basin Institute, Department of Anthropology, Stony Brook University, Stony Brook, New York 11794-4364, USA.

^5^ Research Laboratory for Archaeology & the History of Art, School of Archaeology, University of Oxford, Oxford OX1 3TG, UK.

^6^ Department of Geography, King’s College London, 40 Bush House (North East Wing), Aldwych, London WC2B 4BG, U.K.

^7^ Centre for Quaternary Research, Department of Geography, Royal Holloway University of London, Egham, Surrey, TW20 0EX, U.K.

^8^ SFF Centre for Early Sapiens Behaviour (SapienCE), University of Bergen, Post Box 7805, 5020, Bergen, Norway.

^9^ Extreme Events Research Group, Max Planck Institute for Chemical Ecology, Hans-Knöll-Straße 8, 07745 Jena, Germany.

^10^ Extreme Events Research Group, Max Planck Institute for the Science of Human History, Kahlaische Straße. 10, 07745, Jena, Germany.

^11^ Extreme Events Research Group, Max Planck Institute for Biogeochemistry, Hans-Knöll-Straße 10, 07745 Jena, Germany.

^12^ Department of Archaeology, Max Planck Institute for the Science of Human History, Kahlaische Straße. 10, 07745, Jena, Germany.

^13^ Human Origins and PalaeoEnvironments Research Group, Oxford Brookes University, Oxford, OX3 0BP, U.K.

^14^ Mansfield College, University of Oxford, Mansfield Rd, Oxford, OX1 3TF, U.K.

^15^ Department of Life Sciences, The Natural History Museum, Cromwell Road, London, SW7 5BD, U.K.

^16^ Department of Archaeology, College of Archaeology and Tourism, King Saud University, Riyadh, Saudi Arabia.

^17^ School of Social Science, University of Queensland, Brisbane QLD 4072, Australia.

^18^ Human Origins Program, National Musuem of Natural History, Smithsonian Institution, Washington, D.C., 20560, USA.

* Corresponding Authors: scerri@shh.mpg.de, petraglia@shh.mpg.de

**Supplementary Text**

Geometric Morphometric Analysis

50 handaxes were randomly selected from the assemblage for further study on shape, with the aim to determine whether forms were discrete or on a range of continuous variation (Table S1). 3D models were created from high resolution photographs in Photoscan using photogrammetry. The models were then exported to Landmark in order to create semi-landmarks/outlines. Sixteen semi-landmarks on outlines were created at angles of 22.5 degrees from each model’s centre, which represented the seventeenth semi-landmark (see Fig. S2). Landmark files were exported to MorphoJ where, following a procrustes fit, they were subjected to Canonical Variates or CVA on allometric (size adjusted) datasets (Tables S1-S4). Partial Least Squares (PLS) was conducted on the shape files together with the spatial information recorded using the DGPS in order to determine whether any relationship existed between the spatial location of the handaxes in the sample and their shape. However, no significant relationship was found.

Chronology

Sediment samples for luminescence dating were prepared at Royal Holloway University of London. Briefly, carbonate was removed by immersion in 1.16 M HCl, followed by immersion in H_2_O_2_ to oxidise organic material. The resulting material was sieved to yield the 210-180 µm fraction, and density separated at 2.75 and 2.62 g/cm^3^ to isolate quartz (2.75-2.62 g/cm^3^) and bulk feldspar (<2.62 g/cm^3^). The quartz fraction was purified using a 1 hour 23 M HF etch, followed by immersion in 11 M HCl for a minimum of 12 hours to remove fluoride precipitates. This material was resieved at 125 µm to remove partially dissolved feldspar grains. At the University of Oxford, the bulk feldspar fraction was density separated at 2.58 g/cm^3^ to isolate the potassium (K-) feldspar fraction. Abundant quartz was extracted from all samples, but unfortunately OSL3 and OSL4 only yielded a small amount of K-feldspar.

The environmental dose rate to etched quartz comprises external beta, gamma and cosmic ray components. In addition to these components, unetched K-feldspars also receive an external alpha dose and internal alpha and beta doses. External alpha dose rates were calculated using uranium, thorium and potassium concentrations determined using a combination of thick-source alpha counting (Daybreak Model 583) and beta counting (Risø GM-25-5 low-level beta counting system, ^[45]^. External beta dose rates were calculated using beta counting data rather than isotope concentrations. External gamma dose rates were measured in the field using an EG&G Ortec digi Dart-LF gamma-spectrometer using the “threshold” method. Cosmic ray dose rates were calculated using site location (28°N, 39°E, ~900 m elevation) and present-day sediment burial depths ^[46]^, assuming a sediment overburden density of 1.80 g/cm^3^. Assuming that each grain comprises 12.5 ± 0.5 % potassium and 400 ± 100 ppm rubidium-87, and considering alpha and beta attenuation factor of ^[47]^, and ^[48]^respectively, an internal feldspar dose rate of 0.80 ± 0.07 Gy.ka-1 was included in the dose rate calculations. The mean burial water content of all samples was assumed to be 5 ± 2.5 %. Dose rates to quartz and feldspar are presented in Table S5.

Equivalent doses were measured on the quartz separates at Royal Holloway University of London using a Risø TL/OSL-DA-15 automated dating system ^[49]^ using the single-aliquot regenerative dose method ^[50]^. Twenty-four multigrain aliquots (5 mm diameter) were measured for each sample. Aliquots were rejected where they yielded 1) an IR-depletion ratio >2σ below unity ^[51]^; 2) a recycling ratio inconsistent with unity at >2σ ^[50]^ or 3) the natural luminescence intensity exceeded the maximum level of the growth curve when fitted with a saturating exponential function using Analyst version 4.31.9, termed “oversaturation” hereafter. Using these criteria 5% of aliquots were rejected due to poor recycling ratios, 34% due to poor IR depletion ratios and 11% due to oversaturation. Several previous studies of quartz from the Nefud Desert have reported similarly high IR-depletion ratio test failure rates (e.g. ^[24,52]^). Although only 11% of the aliquots measured were oversaturated, a large number of the accepted aliquots yielded natural luminescence intensities very close to the saturation level of the growth curve. This observation suggests that the samples are at or beyond the maximum measurable age using quartz luminescence. Consequently, radiofluorescence measurements were made on K-feldspar separates from the same samples, since this technique has a considerably larger applicable age range than quartz luminescence.

Equivalent doses were measured for 12 multigrain K-feldspar aliquots (2 mm diameter) from each sample using the infrared-radiofluorescence (IR-RF) single aliquot regenerative (SAR) protocol at high temperature (hereafter RF_70_) ^[53]^ at the University of Oxford. For samples OSL3 and 4, only few aliquots showed an RF signal, indicating that the samples do not contain many feldspars. RF_70_ measurements were carried out on a Freiberg Instruments *Lexsyg Research* reader ^[54]^ equipped with a specially designed ^90^Sr/^90^Y ring-source ^[55]^ delivering 0.057 ± 0.03 Gy.s^-1^. RF_70_ signal detection was made through a Chroma D850/40 interference filter. For bleaching, we used the built-in solar simulator. Equivalent dose were determined using the function analyse IRSAR:RF () in the open source **R** package 'Luminescence' version 0.9.0 ^[56,57]^. Figure S3 shows the results of the RF_70_ measurements for the four samples.

Luminescence ages are presented in Table S5. The quartz samples yield younger ages than their paired feldspar sample. This supports the suggestion that the quartz samples are at/close to saturation, based on visual inspection of individual growth curves, and hence only yield minimum ages. To avoid confusion, we have not presented ages for the quartz samples in Table S5, and instead base the chronology for An Nasim on the RF_70_ data.

**Supplementary references**

45. Bøtter-Jensen, L. & V. Mejdahl. Assessment of beta dose-rate using a GM multicounter system. *Int. J. Radiat. Appl. Instrum.* **D 14**, 187–191 (1988).

46. Prescott, J. R. & Hutton, J. T. Cosmic ray and gamma ray dosimetry for TL and ESR. *Int. J. Radiat. Appl. Instrum.* **D 14**, 223–227 (1988).

47. Brennan, B. J., Lyons, R. G. & Phillips, S. W. Attenuation of alpha particle track dose for spherical grains. *Int. J. Radiat. Appl. Instrum.* **D 18**, (1991).

48. Guérin, G. *et al.* Multi-method (TL and OSL), multi-material (quartz and flint) dating of the Mousterian site of Roc de Marsal (Dordogne, France): Correlating Neanderthal occupations with the climatic variability of MIS 5-3. *J. Archaeol. Sci.* **39**, 3071–3084 (2012).

49. Bøtter-Jensen, L., Andersen, C. E., Duller, G. A. T. & Murray, A. S. Developments in radiation, stimulation and observation facilities in luminescence measurements. *Radiat. Meas.* **37**, 535–541 (2003).

50. Murray, A. S. & Wintle, A. G. Luminescence dating of quartz using an improved single-aliquot regenerative-dose protocol. *Radiat. Meas.* **32**, 57–73 (2000).

51. Duller, G. A. T. Distinguishing quartz and feldspar in single grain luminescence measurements. *Radiat. Meas.* **37**, 161–165 (2003).

52. Petraglia, M. D. *et al.* Hominin Dispersal into the Nefud Desert and Middle Palaeolithic Settlement along the Jubbah Palaeolake, Northern Arabia. *PLoS One* **7**, e49840 (2012).

53. Frouin, M. *et al.* An improved radiofluorescence single-aliquot regenerative dose protocol for K-feldspars. *Quat. Geochronol.* **38**, 13–24 (2017).

54. Richter, D., Richter, A. & Dornich, K. Lexsyg - a new system for luminescence research. *Geochronometria* **40**, 220–228 (2013).

55. Richter, D., Pintaske, R., Dornich, K. & Krbetschek, M. A novel beta source design for uniform irradiation in dosimetric applications. *Anc. TL* **30**, 57–63 (2012).

56. Kreutzer, S. *et al.* Introducing an R package for luminescence dating analysis. *Anc. TL* **30**, 1–8 (2012).

57. Kreutzer, S. *et al.* Luminescence: Comprehensive Luminescence Dating Data Analysis. R package version. https://cran.r-project.org/package=Luminescence (2018). 15.

**Supplementary Figures**

**Fig. S1.**


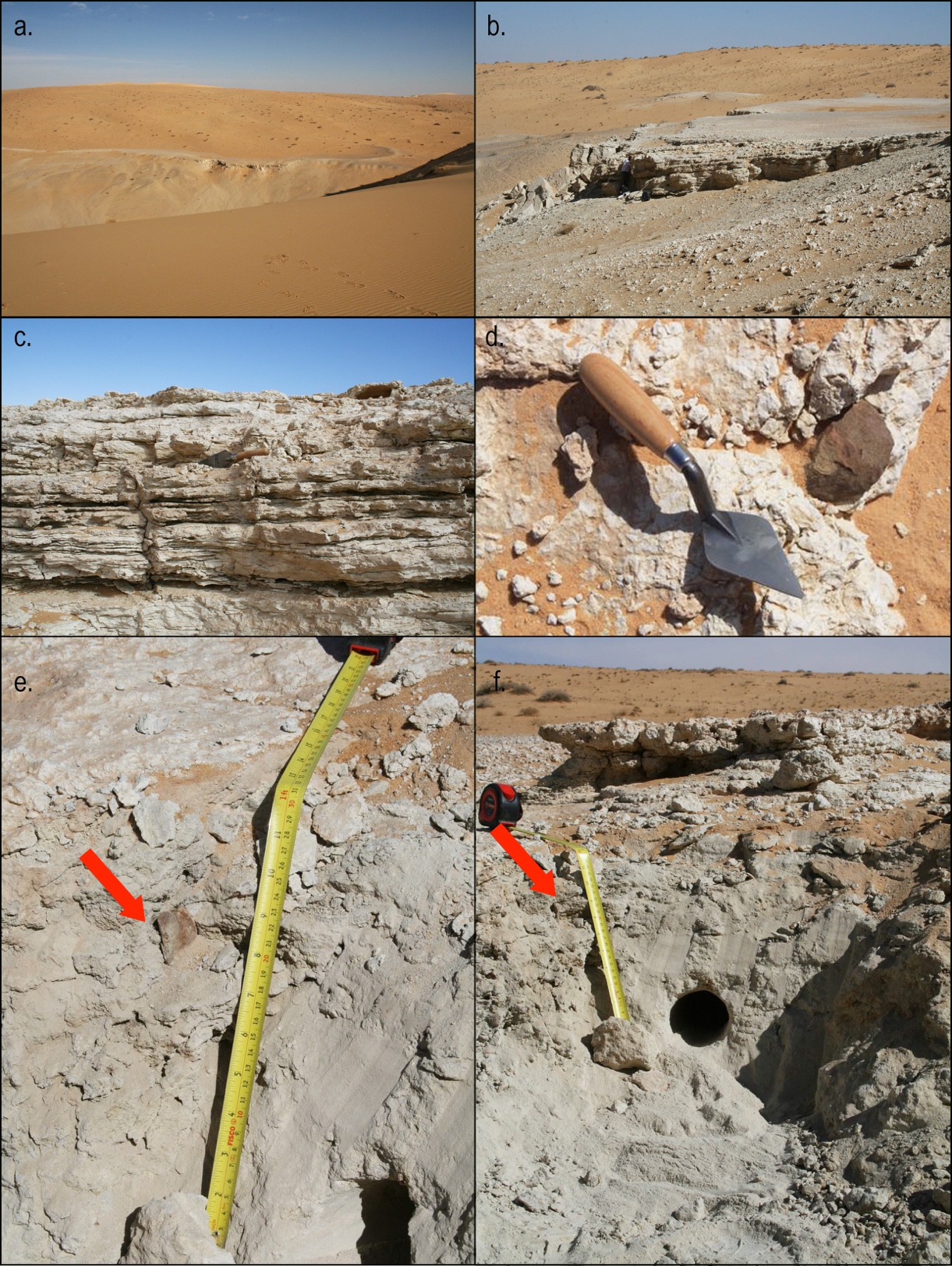


**Fig. S1:** Geomorphological context of An Nasim. a: Nasim basin, showing its partial exposure as its eastern margin is covered by a large dune; b: Marls, which are at their thinnest at the basin margin and thicken towards the centre, with the bedding within the marls dipping towards the centre of the basin; c: Desiccation cracks in the upper part of the sequence; d: base of buried handaxe in the marls, layer 11 ; e: buried thinning flake emerging from sediment sample excavation ; f: location of buried flake in association with NSM1-2017 luminescence sample.

**Fig. S2.**


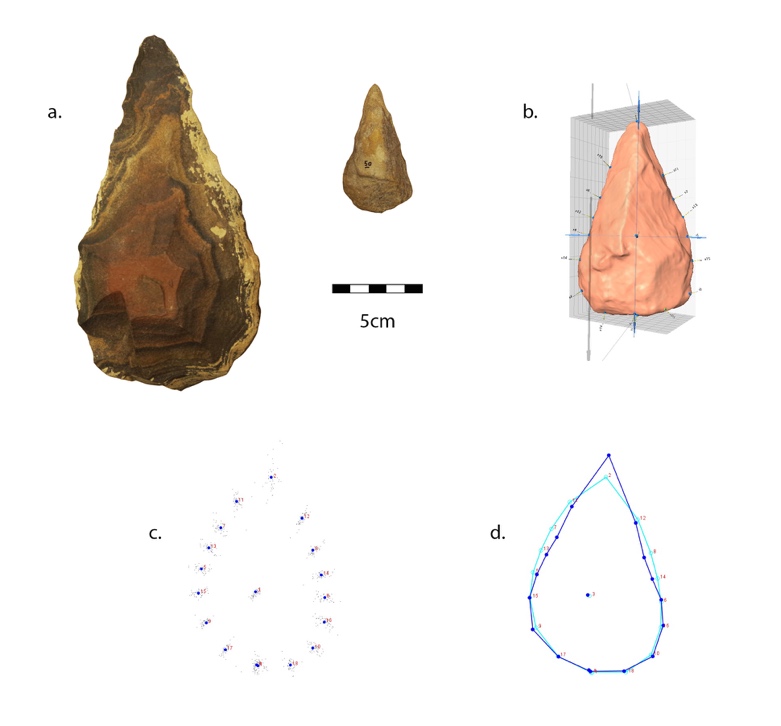


**Fig. S2:** Data processing for analysis. a: two handaxes from An Nasim, showing differences in size; b. photogrammetry model of a handaxe in Landmark; c: Procrustes fit in MorphoJ; d. wire frames.

**Fig. S3**.

| NSM1-OSL3 (Layer 7)  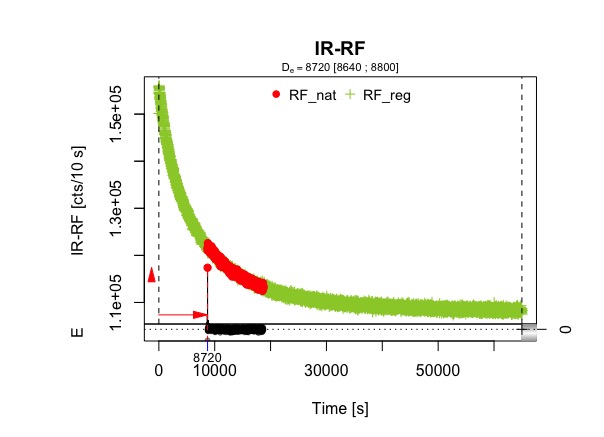 |
| --- |
| NSM1-OSL4 (Layer 8)  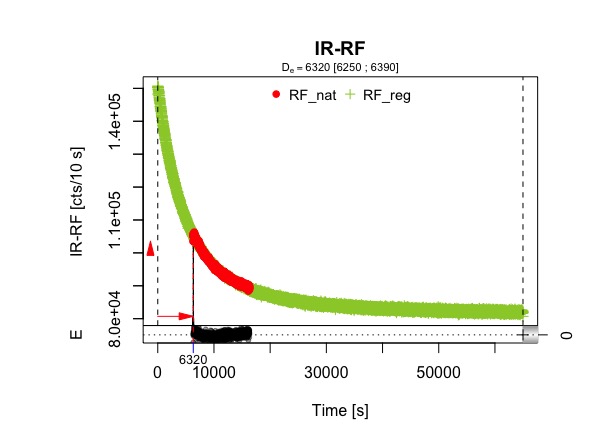 |
| NSM1-2017 (Layer 11)  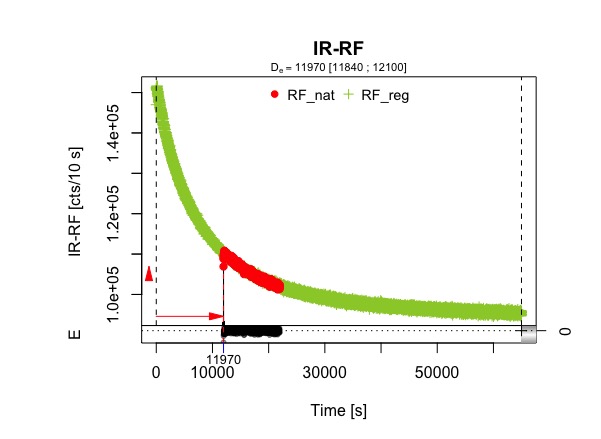 |

**Fig. S3:** Example of RF_70_ curves of one aliquot, the equivalent dose is obtained by sliding the natural signal (red) on the regenerated signal (green), as well as Kernel Density Estimation plot and histogram of equivalent doses.

**Supplementary Tables**

**Table S1.**

| Group 1 | Cordiform | 24 |
| --- | --- | --- |
| Group 2 | Ovate | 10 |
| Group 3 | Triangular | 8 |

Table S1: Handaxe shape group observations

**Table S2.**

| **Eigenvalues** | **% Variance** | **Cumulative %** |
| --- | --- | --- |
| 12.25476608 | 67.698 | 67.698 |
| 5.84729523 | 32.302 | 100.000 |

Table S2: Variation among groups, scaled by the inverse of the within-group variation

**Table S3.**

|  | **Cordiform** | **Ovate** |
| --- | --- | --- |
| **Ovate** | 7.7339 |  |
| **Triangular** | 6.3200 | 9.7642 |

Table S3: Mahalanobis distances among groups.

**Table S4**

|  | **Cordiform** | **Ovate** |
| --- | --- | --- |
| **Ovate** | 0.0531 |  |
| **Triangular** | 0.0820 | 0.1157 |

Table S4. Procrustes distances among groups.

Table S5.

| Sample (NSM1-…) | Mineral^1^ | External dose rates (Gy/ka) | | | | | D_e_ (Gy) | Age (ka)^3^ |
| --- | --- | --- | --- | --- | --- | --- | --- | --- |
|  |  | Alpha | Beta | Gamma | Cosmic | Total^2^ |  |  |
| 2017 | Q | - | 0.17±0.01 | 0.38±0.04 | 0.24±0.02 | 0.79±0.04 | 277±20 | - |
|  | F | 0.011±0.002 | 0.17±0.01 | 0.38±0.04 | 0.24±0.02 | 1.58±0.08 | 520±26 | 330±23 |
| OSL4 | Q | - | 0.45±0.03 | 0.37±0.04 | 0.20±0.01 | 1.02±0.04 | 205±16 | - |
|  | F | 0.021±0.004 | 0.45±0.03 | 0.37±0.04 | 0.20±0.01 | 1.82±0.08 | 442±37 | 243±23 |
| OSL3 | Q | - | 0.38±0.02 | 0.32±0.03 | 0.17±0.00 | 0.87±0.04 | 188±14 | - |
|  | F | 0.019±0.004 | 0.38±0.02 | 0.32±0.03 | 0.17±0.00 | 1.67±0.08 | 517±15 | 310±17 |

Table S5. Dose rate and equivalent dose data for quartz and feldspar separates from An Nasim. Dose rates were calculated in DRAC v 1.2 ^[28]^ using a 5±2.5 % mean moisture content for all samples.

^1^ Q = quartz, F = feldspar.

^2^ The total dose rate for feldspars includes all external dose rate components and an additional 0.80±0.07 Gy/ka internal dose rate contribution.

^3^ The quartz samples yield younger ages than their paired feldspar samples. This is taken as confirming the suggestion, based on visual inspection of individual growth curves, that the quartz samples are at/close to saturation and hence only yield minimum ages. Consequently, to avoid confusion, none of the ages for quartz samples have not been presented here.
